# Free 25-hydroxyvitamin-D concentrations are lower in children with renal transplant compared with chronic kidney disease

**DOI:** 10.1007/s00467-020-04472-z

**Published:** 2020-01-22

**Authors:** Evgenia Preka, Mandy Wan, Karen L Price, David A Long, Helen Aitkenhead, Rukshana Shroff

**Affiliations:** 1 Renal Unit, Great Ormond Street Hospital for Children NHS Foundation Trust, London, UK; 2 Developmental Biology and Cancer Programme, UCL Great Ormond Street Institute of Child Health, London, UK; 3 Institute of Pharmaceutical Science, King’s College London, London, UK; 4 Department of Chemical Pathology, Great Ormond Street Hospital NHS Foundation Trust, London, UK

**Keywords:** 25-hydroxyvitamin D, Chronic kidney disease, Pediatric renal transplantation, Children

## Abstract

**Background:**

Total serum 25-hydroxyvitamin D [25(OH)D] is considered the best marker of vitamin D status and used routinely in clinical practice. However, 25(OH)D is predominantly bound to vitamin D-binding protein (VDBP), and it has been reported that the free-25(OH)D and 25(OH)D loosely bound to albumin fraction correlates better with clinical outcomes.

**Methods:**

We assessed total-25(OH)D, measured free-25(OH)D, and calculated free-25(OH)D and their relationship with VDBP and biomarkers of mineral metabolism in 61 children (22 CKD 2–3, 18 dialysis, and 21 post-transplant).

**Results:**

Total-25(OH)D concentrations were comparable across the three groups (*p* = 0.09), but free- and bioavailable-25(OH)D (free- and albumin-25(OH)D) were significantly lower in the transplant group (both: *p* = 0.01). Compared to CKD and dialysis patients, the transplant group had significantly higher VDBP concentrations (*p* = 0.03). In all three groups, total-25(OH)D concentrations were positively associated with measured free-, calculated free-, and bioavailable-25(OH)D. Multivariable regression analysis showed that total-25(OH)D was the only predictor of measured free-25(OH)D concentrations in the dialysis group (β = 0.9; R^2^ = 90%). In the transplant group, measured free-25(OH)D concentrations were predicted by both total-25(OH)D and VDBP concentrations (β = 0.6, − 0.6, respectively; R^2^ = 80%). Correlations between parathyroid hormone with total-25(OH)D and measured and calculated free-25(OH)D were only observed in the transplant group (all: *p* < 0.001).

**Conclusions:**

In transplanted patients, VDBP concentrations were significantly higher compared to CKD and dialysis patients, and consequently, free-25(OH)D concentrations were lower, despite a comparable total-25(OH)D concentration. We suggest that free-25(OH)D measures may be required in children with CKD, dialysis, and transplant, with further research required to understand its association with markers of mineral metabolism.

## Introduction

Vitamin D deficiency is prevalent in patients with chronic kidney disease (CKD), including kidney transplant recipients, and is considered to contribute to the pathogenesis of CKD-mineral and bone disorder (CKD-MBD) [1–4]. Numerous observational studies have associated low vitamin D status with higher parathyroid hormone (PTH) concentrations [5–8], and observational data have reported an inverse relationship between vitamin D status and multiple adverse health-related outcomes, including decreased bone mineral density [9, 10], faster CKD progression [11, 12], acceleration of vascular calcifications [13], progression of allograft dysfunction [14, 15], and increased risk of cardiovascular morbidity and mortality [16–18]. In addition, there are studies suggesting that vitamin D supplementation can decrease PTH concentrations and significantly delay the time to development of secondary hyperparathyroidism [19, 20]. Recognizing the potential negative effects of low vitamin D status, current clinical practice guidelines provide consensus support for determining vitamin D status and correction of deficiency through vitamin D supplementation in adults and children with CKD [21–23].

Total serum 25-hydroxyvitamin D [total-25(OH)D] concentration is regarded as the most reliable biochemical marker to assess an individual’s nutritional vitamin D status; it reflects vitamin D supply from cutaneous biosynthesis and diet and has the advantage of not being under tight homeostatic control as well as having a long circulating half-life [24, 25]. However, relying on total-25(OH)D concentrations conflicts with the free hormone hypothesis which postulates that the biological activity of hormones is determined by their free (non-protein-bound) concentrations as protein-bound ligands cannot freely diffuse across cell membrane to interact with targets at a cytoplasmic or nuclear level [26]. In fact, around 85–90% of total-25(OH)D circulates in the plasma tightly bound to vitamin D-binding protein (VDBP) with 10–15% bound to albumin with much lower affinity, leaving less than 1% in the free form [free-25(OH)D]. This would imply that variations in VDBP concentrations or its affinity for 25(OH)D will alter the relationship between total-25(OH)D concentrations and its free fraction.

Several studies across different populations, including adults and children, have reported that free-25(OH)D or its bioavailable fraction [free-25(OH)D and albumin bound 25(OH)D] are more strongly associated with markers of mineral metabolism than total-25(OH)D [27–32]. In a study of adult dialysis patients, corrected calcium and PTH concentrations were not associated with total-25(OH)D but correlated with bioavailable-25(OH)D [28]. Similarly, bioavailable-25(OH)D was more strongly associated with PTH than total-25(OH)D in adults with nephrotic syndrome, while bioavailable-25(OH)D concentrations correlated with bone mineral density but not with total-25(OH)D in the same population [30]. In healthy children, Lopez-Molina et al. also reported that measured free-25(OH)D correlated better with markers of phosphocalcic metabolism than total-25(OH)D [32]. However, whether free- or bioavailable-25(OH)D is a better marker remains controversial as other studies have yielded conflicting results where the correlations of total-, free-, and bioavailable-25(OH)D with markers of mineral metabolism did not differ [33–37]. Moreover, different methods for determining VDBP (e.g., monoclonal assay, polyclonal assay, mass spectrometry) and free-25(OH)D concentrations (e.g., indirect estimations using equations, directly measured) further confound interpretation.

Data on free-25(OH)D concentrations in CKD patients is scarce. Available published data in this population have largely been determined by calculations using the Bikle’s formula or a modified Vermeulen equation that relies heavily on the accurate measurement of VDBP and the assumption of a single affinity of VDBP for the vitamin D metabolites [28, 29, 33, 34, 38, 39]. Notably, in the few studies of adults and children with CKD, serum VDBP concentrations were measured using a monoclonal antibody which recognizes a single epitope near the polymorphic region of VDBP and thus show preferential binding to the Gc1S isoform, leading to underestimation of VDBP concentrations that differ by race [28, 33, 34, 40, 41]. The polymorphic isoforms of VDBP have also been reported to differ in their affinities for 25(OH)D, with some reports suggesting that the affinity of VDBP is altered in some clinical conditions including patients with liver disease and during pregnancy [26, 42]. Thus, using a polyclonal enzyme-linked immunosorbent assay (ELISA), which recognizes multiple epitopes on the VDBP and thus unaffected by VDBP isoforms, for quantifying VDBP [41], an ELISA for directly measuring free-25(OH), and using estimated free-25(OH)D as a comparison, the aim of our study was to determine total-25(OH)D, directly measured free-25(OH)D, calculated free-25(OH)D, VDBP, and their relationship with biomarkers of mineral metabolism in children with CKD stage 2–3, on dialysis, and with functioning renal transplant.

## Methods

### Study population

This was a post hoc cross-sectional analysis of 61 pediatric CKD and transplant patients (22 CKD stages 2–3, 18 dialysis, and 21 post-transplant) who attended the Renal Unit at Great Ormond Street Hospital (GOSH) for children. Stored serum samples were obtained from three sources [20, 43, 44]. The ERGO study is a randomized, double-blinded, placebo-controlled trial investigating the effects of ergocalciferol supplementation on the time to development of secondary hyperparathyroidism in children with CKD stages 2–4 without pre-existing hyperparathyroidism [20]; none of the 22 children included in this analysis were taking ergocalciferol at the time the serum sample was taken. Dialysis patients were included from the GOSH participants in the 4C study, a European multicenter prospective observational study following 688 pediatric CKD patients 6–17 years of age with initial eGFR of 10–60 mL/min/1.73 m^2^ [43]. Transplanted patients were included from a cross-sectional study of all children with a functioning renal transplant at GOSH to assess correlations of 25(OH)D levels with key patient level outcomes [44].

### Biochemical analysis

Non-fasting blood samples were collected and centrifuged at 5000 rpm for 10 min at 4 °C. The serum and plasma were stored at − 80 °C until analysis. Routine biochemistry, including PTH and other markers of mineral metabolism, was analyzed at the Department of Chemical Pathology at GOSH. Glomerular filtration rate (GFR) was estimated using the bedside Schwartz formula. PTH concentrations were measured by the Immulite 2000 Intact PTH immunoassay (Siemens Healthcare Diagnostics, Frimley, Surrey, UK).

Total-25(OH)D concentrations were analyzed by isotope-dilution liquid chromatography-tandem mass spectrometry at GOSH (Waters Xevo TQ-S, Waters UK, Elstree, Herts, UK). The inter-assay coefficients of variation (CV) for total-25(OH)D_2_ and total-25(OH)D_3_ were 5.1% at 59 nmol/L and 8.4% at 55 nmol/L, respectively. The intra-assay CVs for total-25(OH)D_2_ and total-25(OH)D_3_ were 6.9% at 19 nmol/L and 6.9% at 39 nmol/L, respectively. ELISA was used for free-25(OH)D (DIASource Future Diagnostics ImmunoAssays, Belgium). The intra-assay and inter-assay CVs range from 4 to 6.3% and 1.9 to 5.5%, respectively. VDBP was also determined by ELISA (Immunodiagnostik AG essay, catalog # K2314, Bensheim, Germany). The intra-assay CV range from 3.2 to 5%, and the inter-assay CV value at a concentration of 19.3 mg/dL was 12.7%.

### Calculation of free- and bioavailable-25(OH)D

We used the formula as shown in Bikle et al. to calculate free-25(OH)D concentration using plasma albumin, serum VDBP concentrations, and total-25(OH)D [38].

$$ free-25(OH)D=\frac{total-25(OH)D}{1+\left(\ {K}_{alb}\times Albumin\right)+\left({K}_{VDBP}\times VDBP\right)} $$

where free-25(OH)D is the concentration of free-25(OH)D in mol/L; K_alb_ is the affinity constant between 25(OH)D and albumin equal to 6 × 10^5^ M^−1^; K_VDBP_ is the affinity constant between 25(OH)D and VDBP equal to 7 × 10^8^ M^−1^; albumin is the concentration of total plasma albumin in mol/L; VDBP is the concentration of total VDBP in mol/L; total-25(OH)D is the concentration of total-25(OH)D in mol/L.

Bioavailable-25(OH)D concentration was calculated as follow:

$$ bioavailable-25(OH)D= free-25\left(\mathrm{OH}\right)\mathrm{D}+ albumin\ bound\ 25(OH)D $$

$$ bioavailable-25\left(\mathrm{OH}\right)\mathrm{D}= free-25\left(\mathrm{OH}\right)\mathrm{D}\times \left({K}_{alb}\times Albumin+1\right) $$

where bioavailable-25(OH)D is concentration of bioavailable-25(OH)D in nmol/L; K_alb_ is affinity constant between 25(OH)D and albumin equal to 6 × 10^5^ M^−1^; albumin is the concentration of total plasma albumin in mol/L.

## Statistical analysis

Distributions of all variables were assessed for normality. Descriptive statistics are presented for demographic and clinical characteristics of the study cohort. Continuous variables are presented as medians and interquartile ranges (IQR), whereas frequencies and percentages are used for categorical variables. Anthropometric indices are expressed as absolute values as well as standard deviation score (SDS) for age and gender. Comparisons of continuous variables between groups were performed using Mann-Whitney or Kruskal-Wallis tests as appropriate. Categorical variables were analyzed using chi-square test. The relationships between continuous variables were assessed with Spearman’s correlation coefficient. Bland-Altman plots were used to assess the agreement between measured-free-25(OH)D and calculated-free-25(OH)D concentrations, with limits of agreement determined by nonparametric method. [45] Multivariable linear regression analysis was conducted with natural log transformed measured free-25(OH)D as the dependent variable, and total-25(OH)D and VDBP concentrations (both natural log transformed) were included as covariates. For all analyses, *P* < 0.05 was considered to be statistically significant. All statistical analyses were performed using Stata 15 (Stata Corp, College Station, Texas).

## Results

### Participant characteristics

Table 1A provides a summary of the demographics and clinical characteristics of the 61 participants. Gender and ethnicity distribution were comparable between the CKD 2–3, the dialysis, and the transplant groups. The median age of the transplant group was significantly older than children with CDK 2–3 (*p* < 0.001) but comparable to children on dialysis (*p =* 0.052). Children with kidney transplant had higher body mass index compared to children with CKD 2–3 (*p* = 0.002) and those on dialysis (*p* = 0.009). The three groups differed with regard to underlying renal diseases; glomerular disease was present in 0% of the CDK 2–3 group, 16.7% of the dialysis group, and 14.3% of the transplant group. Of the eight children with glomerular disease or Wilms’ tumor, seven had bilateral nephrectomies prior to the study (Table 1A). There were no differences in systolic blood pressure SDS between groups.

**Table 1:** Participant characteristics

	CKD 2–3 (*n* = 22)	Dialysis (*n* = 18)	Transplant (*n* = 21)	*p*^a^
A				
Age, year	8.9 (3.6, 12.6)	13.8 (6.3, 14.5)	14.3 (11.2, 16.1)	< 0.001
Male, n (%)	10 (45.5)	7 (38.9)	10 (47.6)	0.85
Race, n				
White/Asian/Black/Mixed/Other	16/3/2/0/1	8/4/5/0/1	14/6/1/0/0	0.24
Anthropometry				
Height, cm	129.1 (97.9, 151)	138.2 (110.8, 158)	149.2 (138.6, 162)	0.02
Height SDS	− 0.44 (− 1.03, 0.60)	− 0.99 (− 2.89, − 0.22)	− 1.04 (− 1.88, − 0.09)	0.17
Weight, kg	28.3 (15.0, 40.7)	36.5 (19, 47)	47 (36.5, 60.9)	0.002
Weight SDS	− 0.07 (− 0.59, 0.43)	− 0.68 (− 1.83, − 0.03)	− 0.11 (− 0.87, 0.76)	0.11
Body mass index	17.2 (16.3, 19.9)	18.2 (16.5, 19.1)	20.5 (19.0, 25.5)	0.004
Underlying renal diagnosis, n (%)				< 0.001
CAKUT	20 (90.9)	6 (33.3)	12 (57.1)	
Cystic kidney disease	0	1 (5.6)	6 (28.6)	
Glomerular disease	0	3 (16.7)	3 (14.3) ^b^	
Wilms’ tumor	1 (4.5)	1 (5.6)	0	
Others	1 (4.5)	3 (16.7)	0	
Unknown	0	4 (22.2)	0	
Time since transplant, months			16.2 (7.5, 20.8)	
Dialysis: PD/HD, n		4/14		
Systolic blood pressure				
mmHg	93 (84, 104)	102 (95, 124)	108 (102, 114)	0.003
SDS	− 0.22 (− 1.42, 0.36)	0.62 (− 0.62, 1.91)	0.19 (− 0.5, 0.57)	0.06
B				
eGFR, ml/min/1.73 m^2^	69 (63.1, 79.2)		61.3 (52.9, 85.5)	0.44^c^
Urea, mmol/L	6.7 (5.7, 7.6)	19.05 (16.6, 22.4)	7.4 (6.2, 8.3)	< 0.001
Albumin, g/L	45 (43, 46)	39 (38, 41)	42 (39, 43)	< 0.001
Calcium (corrected), mmol/L	2.28 (2.25, 2.35)	2.42 (2.36, 2.55)	2.36 (2.27, 2.41)	0.002
Parathyroid hormone, pmol/L	3.6 (2.9, 4.3)	11.5 (5.5, 37.3)	5.1 (3.5, 8.4)	0.001
Phosphate, mmol/L	1.49 (1.35, 1.65)	1.61 (1.29, 1.8)	1.3 (1.14, 1.41)	0.002
Alkaline phosphatase, U/L	200 (163, 233)	167 (124, 238)	177 (95, 262)	0.71
Urine Albumin: Creatinine, mg/mmol	1.6 (0.8, 4.6)^d^		7.1 (3.5, 26.2)^e^	0.007^c^
Medications, n (%)				
Colecalciferol	0	9 (50)	1 (4.8)	< 0.001
Alfacalcidol	0	17 (94.4)	5 (23.8)	< 0.001
Calcium-based phosphate binder	5 (22.7)	16 (88.9)	0	< 0.001
Sevelamer	0	1 (5.6)	0	0.3
Cinacalcet	0	0	0	
ACEi/ARB	3 (13.6)	5 (27.8)	0	0.04
Prednisolone	0	2 (11.1)	20 (95.2)	< 0.001
Tacrolimus	0	2 (11.1)	21 (100)	< 0.001

Data are presented as number with the percentage in parenthesis or as median and interquartile range in parenthesis. *ACEi* angiotensin-converting-enzyme inhibitor, *ARB* angiotensin-receptor blocker, *CAKUT* congenital anomalies of the kidney and urinary tract, *CKD* chronic kidney disease, *HD* hemodialysis, *PD* peritoneal dialysis, *SDS* standard deviation score

^a^Kruskal-Wallis or chi-square test

^b^1 child did not have bilateral nephrectomy

^c^Mann-Whitney test between CKD 2–3 and transplant groups

^d^*n* = 20

^e^*n* = 13

Laboratory parameters and concomitant medications are summarized in Table 1B. Children with CKD 2–3 had comparable eGFR measurements to those with transplants. All children, apart from one on dialysis, had plasma albumin concentrations between 35 and 50 g/L, but comparison across the three groups showed lowest concentrations in the dialysis group (*p* < 0.001). In addition, plasma albumin concentrations were significantly lower in children with kidney transplant when compared to children with CKD 2–3 (*p* < 0.001) but comparable to children on dialysis (*p =* 0.12). Of the 33 children with results for urine albumin-creatinine ratio, four children had albuminuria > 30 mg/mmol; albumin-creatinine ratio was significantly higher among the transplant recipients than children with CKD 2–3. Due to the cross-sectional design of the study, the number of patients taking colecalciferol and alfacalcidol reflected routine clinical care with a larger proportion of patients taking colecalciferol and alfacalcidol in the dialysis group (Table 1B).

### Serum concentrations of 25(OH)D metabolites, VDBP, and CKD-MBD measures

**Fig. 1:**
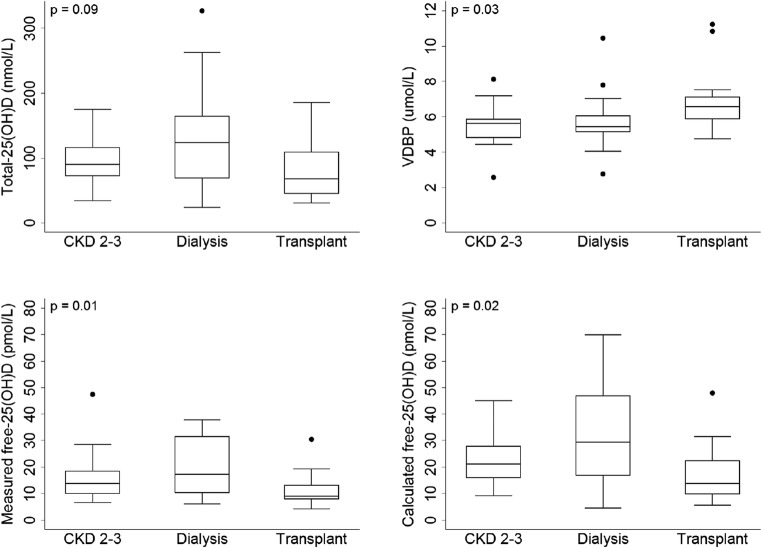
Serum concentrations of total 25-hydroxyvitamin D (25(OH)D), vitamin D-binding protein (VDBP), measured and calculated free-25(OH)D by CKD stages. Between groups of *p* values (Kruskal-Wallis). Mann Whitney *p* values: [CKD 2–3 vs. dialysis: total-25(OH)D: *p* = 0.19; VDBP: *p* = 0.81; measured free-25(OH)D *p* = 0.27; calculated free-25(OH)D *p* = 0.18]; [CKD 2–3 vs. transplant: Total-25(OH)D: *p* = 0.15; VDBP: *p* = 0.01; measured free-25(OH)D *p* = 0.01; calculated free-25(OH)D *p* = 0.02]; [dialysis vs. transplant: total-25(OH)D: *p* = 0.06; VDBP: *p* = 0.04; measured free-25(OH)D *p* = 0.01; calculated free-25(OH)D *p* = 0.03]

Serum concentrations of total-25(OH)D were comparable across the three groups (*p* = 0.09), but measured free-, calculated free- and bioavailable-25(OH)D concentrations in the transplant group were significantly lower than the other two groups (measured free-25(OH)D: *p* = 0.01; calculated free-25(OH)D: *p* = 0.02; bioavailable-25(OH)D; *p* = 0.01) (Fig. 1 and Supplementary Table 1). VDBP concentrations also differed significantly across the three groups (*p* = 0.03), with the transplant group having higher concentrations compared to those with CKD 2–3 and on dialysis (Fig. 1 and Supplementary Table 1). Despite a higher albumin-creatinine ratio, suggestive of greater urinary protein loss, the transplant group showed higher serum VDBP concentrations than the CKD 2–3 group (*p* = 0.01). Comparison between children with CKD 2–3 and those on dialysis showed no significant difference in any of the 25(OH)D measures or VDBP concentrations. Conversely, in the transplant group, all parameters except total-25(OH)D differed significantly when compared to either CKD 2–3 (all *p* < 0.02) or dialysis group (all *p* < 0.05).

### Comparison between directly measured and calculated free-25(OH)D concentrations

**Fig. 2:**
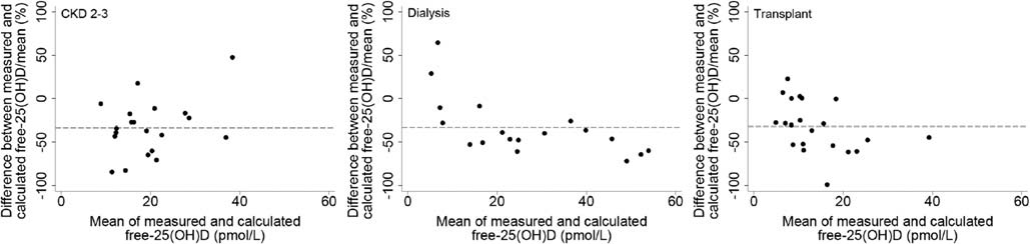
Bland-Altman plots between measured and calculated free-25-hydroxyvitamin D (25(OH)D) by CKD stages. Horizontal dotted lines present mean percentage difference. CKD 2–3: *n* = 20 (unable to calculate free-25(OH)D concentration due to no VDBP data for one patient and no total-25(OH)D data for another); dialysis *n* = 18; transplant: *n* = 2.

The measured free-25(OH)D concentrations were lower than their respective calculated values; the mean differences were − 5.64, − 12.11, and − 5.47 pmol/L for children with CKD 2–3, on dialysis, and renal transplant, respectively. The limits of agreement (5th and 95th percentiles) were wide: − 15 to 3 pmol/L for CKD 2–3, − 33.5 to 1.5 pmol/L for dialysis, and – 16.3 to 0.4 pmol/L for transplants. Figure 2 shows the Bland-Altman plots between measured and calculated free-25(OH)D concentrations expressed as percentage differences. The mean percent bias was approximately − 32% across all three groups, and the percent bias increased with increasing free-25(OH)D concentration.

### Correlates of different forms of 25(OH)D and VDBP

In all three groups, total-25(OH)D concentrations were positively associated with measured free-, calculated free-, and bioavailable-25(OH)D concentrations (Table 2). There was no association between VDBP and total-25(OH)D concentrations in any of the groups (Table 2). VDBP concentrations were negatively associated with measured free-, calculated free-, and bioavailable-25(OH)D in the dialysis and transplant groups only (Table 2). In the dialysis group, weight was found to correlate negatively with all the 25(OH)D concentrations, but this was not observed in the other two groups (Table 2). In addition, weight-adjusted dosage of colecalciferol was found to correlate positively with total 25(OH)D (r = 0.65, *p* = 0.003), measured free-25(OH)D (r = 0.57, *p* = 0.01), calculated free-25(OH)D (r = 0.62, *p* = 0.006), and bioavailable-25(OH)D (r = 0.55, *p* = 0.02) in the dialysis group.

**Table 2:** Correlations of serum concentrations of different forms of 25-hydroxyvitamin D (25(OH)D), vitamin D-binding protein, albumin, weight, and chronic kidney disease-mineral and bone disease (CKD-MBD) measures by CKD stages

	Total 25(OH)D	Bioavailable 25(OH)D	Free (measured) 25(OH)D	Free (calculated) 25(OH)D	VDBP
CKD 2–3 (*n* = 22)					
Bioavailable 25(OH)D	0.74**				
Free (measured) 25(OH)D	0.77**	0.98**			
Free (calculated) 25(OH)D	0.87**	0.74**	0.73**		
VDBP	0.13	− 0.03	0.11	− 0.29	
Albumin	− 0.54**	− 0.13	− 0.20	− 0.45**	− 0.15
Calcium (Cor)	0.31	0.10	0.16	0.14	0.51**
Phosphate	0.14	0.08	0	0.29	− 0.21
PTH	− 0.09	0	− 0.01	0.03	− 0.25
Weight	− 0.40	− 0.30	− 0.26	− 0.34	− 0.16
Dialysis (*n* = 18)					
Bioavailable 25(OH)D	0.94**				
Free (measured) 25(OH)D	0.92**	0.96**			
Free (calculated) 25(OH)D	0.94**	0.95**	0.96**		
VDBP	− 0.35	− 0.41*	− 0.47**	− 0.53**	
Albumin	0.15	0.25	0.12	0.24	− 0.24
Calcium (Cor)	0.08	0.02	0.11	0.11	0
Phosphate	− 0.27	− 0.33	− 0.29	− 0.38	− 0.07
PTH	− 0.13	− 0.10	0	− 0.08	− 0.30
Weight	− 0.46*	− 0.52**	− 0.57**	− 0.52**	0.09
Transplant (*n* = 21)					
Bioavailable 25(OH)D	0.67**				
Free (measured) 25(OH)D	0.64**	0.98**			
Free (calculated) 25(OH)D	0.94**	0.79**	0.79**		
VDBP	− 0.14	− 0.59**	− 0.65**	− 0.42*	
Albumin	− 0.14	0.08	− 0.02	− 0.19	0.22
Calcium (Cor)	− 0.19	0.02	0.08	− 0.08	− 0.07
Phosphate	− 0.07	− 0.06	− 0.05	− 0.04	0.11
PTH	− 0.71**	− 0.73**	− 0.72**	− 0.74**	0.16
Weight	− 0.04	− 0.23	− 0.30	− 0.16	0.36

Data represent spearman correlation. * *p* < 0.1; ** *p* < 0.05

In multivariable regression analysis with VDBP and total-25(OH)D concentrations as covariates, total-25(OH)D concentrations remained the only predictor of measured free-25(OH)D concentrations in the dialysis group (Table 3). On the other hand, both lower total-25(OH)D concentrations and higher VDBP concentrations were independently associated with lower measured free-25(OH)D concentrations in the transplant group (Table 3).

**Table 3:** Multivariable regression analysis for correlates of measured free-25-hydroxyvitamin D (25(OH)D) concentration by chronic kidney disease (CKD) stages

	β	*p*
Dialysis (adjusted R^2^ = 0.90) Predictors		
log total-25(OH)D, nmol/L	0.90	< 0.001
log VDBP, μmol/L	− 0.14	0.11
Transplant (adjusted R^2^ = 0.80) Predictors		
log total-25(OH)D, nmol/L	0.63	< 0.001
log VDBP, μmol/L	− 0.60	< 0.001

### Correlation with CKD-MBD measures

Among children with CKD 2–3, albumin correlated with total-25(OH)D concentrations (r = − 0.54, *p* < 0.05) and calculated free-25(OH)D concentrations (r = −0.45, *p* < 0.05). Corrected calcium concentrations were found to correlate positively with VDBP concentrations (r = 0.51, *p* < 0.05). There were no associations between measured free-25(OH)D concentrations and any of the CKD-MBD measures in this group.

In the dialysis group, there were also no significant correlations between any of the 25(OH)D concentrations and CKD-MBD measures. PTH was inversely associated with total-, directly measured free-, calculated free-, and bioavailable-25(OH)D in the transplant group only (Table 2).

## Discussions

In this study, we have shown that despite having comparable total-25(OH)D concentrations, VDBP concentrations were significantly higher in the transplanted group with lower measured free-25(OH)D concentrations, as compared to children with early CKD and those on dialysis. VDBP concentrations remained independently associated with lower free-25(OH)D concentrations in transplanted patients, after adjusting for total-25(OH)D, whereas this relationship was absent in the CKD (despite comparable eGFR) and dialysis groups. Measuring total-25(OH)D concentrations in pediatric CKD patients might be misleading, particularly in renal transplant recipients, as the total-25(OH)D does not reflect the unbound form of the circulating vitamin and may overestimate the free (and bioavailable) part of the hormone. These observations suggest that consideration of VDBP and its effects on free-25(OH)D concentrations may better inform our understanding of the differences in MBD among children with CKD and renal transplant.

Our data question whether current clinical practice of measuring total-25(OH)D is the most reliable biomarker of vitamin D status. A number of other studies have raised questions about the interpretation of total-25(OH)D concentrations in diseases or conditions in which VDBP or albumin synthesis is affected [26]. In our cohort, all but one patient had plasma albumin concentrations in the normal range. However, 20/21 transplant patients were on steroid therapy, and all received calcineurin inhibitors (CNIs), both of which could alter the relationship between free- and total-25(OH)D concentrations [26, 46]. The use of steroids has been associated with an increase in VDBP expression with oral contraceptive administration and in children with severe therapy-resistant asthma [26, 47]. In addition, reports of steroids induced up-regulation of 24-hydroxylase activity in experimental models would suggest increased degradation of 25(OH)D and 1,25(OH)_2_D, which support observations that glucocorticoid use is associated with lower total-25(OH)D concentrations [48–51]. While the cross-sectional design of these analyses and our data do not permit a clear causal inference, it is of interest to note that recent studies including ours have reported a strong positive correlation between free- and total-25(OH)D [26, 52]. The effects of CNIs on cytochrome P450 (CYP) expression, and subsequently 25(OH)D and 1,25(OH)_2_D concentrations, are less clear. In experimental setting, CNIs displayed CYP3A4 inhibitory effects and increased synthesis of 1,25(OH)_2_D_3_ [46, 53]. 1,25(OH)_2_D, on the other hand, has been shown to exhibit CYP3A4-inducing properties, with a growing number of studies suggesting a strong link between CYP3A4 and its catabolic activity toward vitamin D metabolites [46, 53]. The complex interplay between these elements to the post-transplant clinical picture is unknown but would appear to all to contribute negatively to bone health.

In the context of renal diseases, an additional consideration is the impact of a patient’s underlying renal disease on VDBP. VDBP- and albumin-bound vitamin D are filtered through the glomeruli, reabsorbed by megalin in the proximal tubule, and undergo subsequent intracellular conversion of 25(OH)D to biologically active 1,25(OH)_2_D [54]. All acute and chronic kidney diseases that are characterized by tubular damage are associated with urinary loss of these protein-bound 25(OH)D resulting in a reduction in serum VDBP, albumin, and total-25(OH)D concentration [26]. However, contrary to patients with liver diseases or in pregnancy, where free-25(OH)D often remains unchanged despite changes in VDBP and total-25(OH)D concentrations, the situation would appear to be more complex in patients with renal diseases. Denburg et al. found that in a cohort of children with CKD stages 2–5D, serum albumin, and total- and free-25(OH)D were lower with more advanced CKD and glomerular diagnoses, but there were no differences in serum VDBP [33]. They subsequently conducted a subgroup analysis and reported that VDBP concentrations were significantly lower in those with glomerular diagnoses compared to all other diagnoses among pre-dialysis patients, with no differences observed in patients on dialysis [33]. On the other hand, Prytula et al. reported that despite urinary and dialysate losses of VDBP and albumin in children on peritoneal dialysis, both serum VDBP and total-25(OH)D concentrations were comparable to pre-dialysis children [55]. In a more recent study of adults CKD patients, the authors reported that free-25(OH)D is not influenced by the level of renal function, although they eluded to a reduction in the ratio of free- or total-25(OH)D in advanced CKD [56]. Taken together, these data suggest that there are a number of factors unique to patients with CKD and renal transplant which could alter the relationship between total and free-25(OH)D concentrations, and thus measuring free-25(OH)D may provide a better index of vitamin D status in this patient population.

Quantification of free-25(OH)D concentrations can either be by direct measurement or calculated based on total-25(OH)D, VDBP, and plasma albumin concentrations [38, 39]. Our study showed that the measured free-25(OH)D concentrations were not in agreement with the calculated free-25(OH)D and were consistently lower than their respective calculated values by approximately − 32% across all three groups with a greater divergence as free-25(OH)D concentrations increased. In line with our results, Toldy et al. concluded that direct measured free-25(OH)D is a more reliable marker for estimation of vitamin D status in a cohort of 100 adult patients with CKD [57]. Notably, the main weakness of using the calculated formula, as highlighted in a recent review by Tsuprykov et al., is its relative inaccuracy arising from the different elements within the formula [58].

First, calculation of free-25(OH)D is largely dependent on the accuracy of total-25(OH)D, albumin, and VDBP measurements. For the latter, genetic variants in VDBP gene mean that measuring VDBP concentrations by a monoclonal antibody ELISA can lead to spuriously low VDBP concentrations in black compared to white subjects [59–61], and the same was reported in Asian population [62]. Monoclonal antibody ELISA that recognizes a single epitope near the polymorphic region of VDBP shows preferential binding to the Gc1 s isoform of VDBP that are more commonly associated with European and poorer recognition of the Gc1f phenotype which predominates in individuals of African ancestry [26, 29]. This was recently demonstrated by Denburg et al. who compared serum VDBP concentrations measured by different methods and found that, in contrast to the monoclonal ELISA results, VDBP concentrations quantified by both the polyclonal ELISA and liquid chromatography-tandem mass spectrometry (LC-MS/MS) did not differ by race [41]. However, it should be noted that the polyclonal ELISA yielded consistently and similarly higher measurements of VDBP, irrespective of genotype, with a median percent difference from the LC-MS/MS-based measurement of 33 to 65% [41]. The second dependent factor for accurately calculating free-25(OH)D concentrations relates to the binding constants for VDBP and albumin which have been suggested to vary depending on various physiologic and pathologic conditions [29]. Intrinsically, the amino acid differences among VDBP polymorphic alleles affect their glycosylation patterns, which in turn alter their isoelectric points and contribute to the differences in their affinity to 25(OH)D [26, 42]. Reduction in VDBP-binding affinity has also been reported in pregnant women [29]. Moreover, albumin-binding affinity is affected by a multitude of biochemical changes, including the accumulation of uremic toxins and acid-base disturbances that occur in patients with CKD [63]. Thus, it seems evident that the use of a single assumed affinity constant would limit the accuracy to which free-25(OH)D concentrations can be calculated.

Overall, studies of VDBP and free-25(OH)D concentrations in CKD patients are limited. Using a monoclonal ELISA, Denburg and colleagues quantified VDBP concentrations in a cohort of pediatric CKD patients and reported a median VDBP concentration of 3.4 μmol/L [33]. Their reported values were comparatively lower than our findings (5.6 μmol/L), but this is in keeping with the observation that monoclonal ELISA, in comparison to polyclonal assay, underestimates VDBP concentrations [41].

In a study of 26 children with CKD stages 4–5D, median VDBP concentration, as determined by a polyclonal assay, was found to be 5.5 μmol/L [55]. Similar result was reported from Denburg et al. who quantified VDBP concentrations in a cohort of adult CKD patients using the same polyclonal assay [41]. They reported a median VDBP concentration of 6.4 μmol/L (IQR: 5.8, 7.2) with a median free-25(OH)D concentration of 15 pmol/L as determined by direct measurement [41]. Further data on directly measured free-25(OH)D concentration in adults CKD patients have also recently been described with reported concentrations of 14.8 ± 5.1 and 16 ± 5 pmol/L in two preliminary reports, respectively [56, 57]. Our findings, which are based on the polyclonal VDBP assay and directly measuring free-25(OH)D, are comparable with these earlier studies and extend the data to children with early stages of CKD and renal transplant.

We acknowledge several limitations of this study. Small patient numbers and the cross-sectional design have limited our ability to examine the effects, if any, of vitamin D metabolites on MBD measures and longitudinal changes in vitamin D status and MBD-related factors. It is possible that missing data on urine albumin-creatinine ratio in the transplant group may have affected our analyses, although a lower median albumin-creatinine ratio would better support the higher serum VDBP concentrations as observed in this group. We were also limited by the lack of a control group and have used the manufacturers’ recommended values as normal range. However, we have attempted to limit our interpretation of the data and the potential directions of associations within the context of the published literature and emerging data from other studies.

In conclusion, this is the first study measuring and comparing total-25(OH)D, measured free-25(OH)D, calculated free-25(OH)D, and VDBP in three distinctive pediatric renal patient groups: CKD, dialysis, and transplant populations. We have shown that despite comparable total-25(OH)D concentrations, serum VDBP, and free-25(OH)D concentrations differ among children with CKD, particularly in the transplanted group, who presented significantly higher VDBP concentrations and lower measured free-25(OH)D concentrations. Our study questions the current practice of determining vitamin D status by measuring total-25(OH)D concentrations. Further research is required to study the association of free-25(OH)D with markers of mineral metabolism, especially in pediatric renal transplant recipients, in order to determine which vitamin D metabolite is biologically relevant and impacts key patient level outcomes.
